# Latent-Class Methods to Evaluate Diagnostics Tests for *Echinococcus* Infections in Dogs

**DOI:** 10.1371/journal.pntd.0002068

**Published:** 2013-02-14

**Authors:** Sonja Hartnack, Christine M. Budke, Philip S. Craig, Qiu Jiamin, Belgees Boufana, Maiza Campos-Ponce, Paul R. Torgerson

**Affiliations:** 1 Section of Veterinary Epidemiology, Vetsuisse Faculty, University of Zurich, Zurich, Switzerland; 2 Department of Veterinary Integrative Biosciences, Texas A&M University, College Station, Texas, United States of America; 3 Institute of Parasitology, Vetsuisse Faculty, University of Zurich, Zurich, Switzerland; 4 Cestode Zoonoses Research Group, School of Environment and Life Sciences, University of Salford, Salford, United Kingdom; 5 Division of Parasitic Diseases, Sichuan Center for Disease Control and Prevention, Chengdu, Sichuan, China; 6 Theoretical Biology, Faculty of Earth and Life Sciences, Amsterdam, The Netherlands; University of Queensland, Australia

## Abstract

**Background:**

The diagnosis of canine echinococcosis can be a challenge in surveillance studies because there is no perfect gold standard that can be used routinely. However, unknown test specificities and sensitivities can be overcome using latent-class analysis with appropriate data.

**Methodology:**

We utilised a set of faecal and purge samples used previously to explore the epidemiology of canine echinococcosis on the Tibetan plateau. Previously only the purge results were reported and analysed in a largely deterministic way. In the present study, additional diagnostic tests of copro-PCR and copro-antigen ELISA were undertaken on the faecal samples. This enabled a Bayesian analysis in a latent-class model to examine the diagnostic performance of a genus specific copro-antigen ELISA, species-specific copro-PCR and arecoline purgation. Potential covariates including co-infection with *Taenia*, age and sex of the dog were also explored. The dependence structure of these diagnostic tests could also be analysed.

**Principle findings:**

The most parsimonious result, indicated by deviance-information criteria, suggested that co-infection with *Taenia* spp. was a significant covariate with the *Echinococcus* infection. The copro-PCRs had estimated sensitivities of 89% and 84% respectively for the diagnoses of *Echinococcus multilocularis* and *E. granulosus*. The specificities for the copro-PCR were estimated at 93 and 83% respectively. Copro-antigen ELISA had sensitivities of 55 and 57% for the diagnosis of *E. multilocularis* and *E. granulosus* and specificities of 71 and 69% respectively. Arecoline purgation with an assumed specificity of 100% had estimated sensitivities of 76% and 85% respectively.

**Significance:**

This study also shows that incorporating diagnostic uncertainty, in other words assuming no perfect gold standard, and including potential covariates like sex or *Taenia* co-infection into the epidemiological analysis may give different results than if the diagnosis of infection status is assumed to be deterministic and this approach should therefore be used whenever possible.

## Author Summary

Dogs are a key definitive host of *Echinococcus* spp; hence, accurate diagnosis in dogs is important for the surveillance and control of echinococcosis. A perfect diagnostic test would detect every infected dog (100% sensitivity) whilst never giving a false positive reaction in non-infected dogs (100% specificity). Since no such test exists, it is important to understand the performance of available diagnostic techniques. We used the results of a study that used three diagnostic tests on dogs from the Tibetan plateau, where there is co-endemicity of *E. granulosus* and *E. multilocularis*. In this study opro-antigen ELISA and copro-PCR diagnostic tests were undertaken on faecal samples from all animals. The dogs were also purged with arecoline hydrobromide to recover adult parasites as a highly specific but relatively insensitive third diagnostic test. We used a statistical approach (Bayesian latent-class models) to estimate simultaneously the sensitivities of all three tests and the specificities of the copro-antigen and copro-PCR tests. We also analysed how some determinants of infection can affect parasite prevalence. This approach provides a robust framework to increase the accuracy of surveillance and epidemiological studies of echinococcosis by overcoming the problems of poor diagnostic test performance.

## Introduction

An efficient and adequate diagnosis is at the core of effective surveillance, control and elimination programmes. For the effectiveness of such programmes, knowledge about test accuracies is indispensable, since even very accurate diagnostic tests might occasionally provide false positive and false negative test results. To diagnose canine echinococcosis, a number of tests are used including arecoline purgation, copro-antigen tests and detection of the presence of the parasite using a PCR analysis of the faeces (reviewed by [1]). Arecoline purgation, a well-established technique of high specificity, has frequently been used in the past. However, it is a laborious and potentially hazardous procedure and has been reported to show poor sensitivity [2]. Hence, alternative methods have been developed for the routine diagnosis of *Echinococcus* infection in dogs and other canids. These tests include copro-antigen ELISA and copro-PCR techniques, but they cannot be considered a gold standard. Only a necropsy of dogs followed by the sedimentation and parasite-counting technique can be considered close to a perfect gold standard, i.e., a gold standard with 100% sensitivity and 100% specificity. However, due to ethical reasons, this procedure cannot be used on the routine surveillance of dogs, since it would involve killing of a large number of affected as well as non-affected dogs. Even on a smaller scale, sacrificing dogs for the purpose of diagnostic test evaluation would potentially be impossible in a Buddhist country.

In the absence of a perfect gold standard in surveillance studies, the accuracy of new or alternative tests cannot be estimated robustly and without bias by comparing such test results of new or alternative results against an imperfect gold standard. For example, in the case of a well-established test with a sensitivity of less than 100%, samples which are falsely classified as negative by such a gold standard test, might be correctly detected as positive by a more sensitive alternative test, thus leading to a biased –in this case too low- estimate of the specificity of the alternative test. Given the absence of a perfect gold standard, however, test accuracies can be estimated robustly using latent-class analysis [3]. In this context, *latent* refers to the idea that the true disease status for each animal is unknown and needs to be estimated from the data. Hui and Walter proposed a model in which two tests with unknown test accuracies are applied to individuals from two populations to estimate sensitivities and specificities as well as prevalences. Their model can be extended to any combination of tests (R) and populations (S) as long as the condition of S≥R/(2^R-1^ – 1) is satisfied [3]. The Hui-Walter model relies on several assumptions, which if violated, may result in unreliable estimates [4]. The first assumption is that the tested individuals are divided into two or more populations with different prevalences. The second assumption is that sensitivities and specificities are constant across different populations. The third assumption is that test results are conditionally independent given the true disease status.

*Echinococcus* infections in dogs vary in parasite abundance and/or prevalence with age [5]. Diagnostic tests which are based on the detection of the parasite might be correlated if the number of parasites found affects the sensitivities of these tests [6]. Although covariance terms for conditional test dependency in the Hui-Walter latent-class model [7]–[10], have been included in a number of analyses that used a Bayesian approach, models using covariates to adjust for factors which might affect the sensitivity and specificity in different populations are scarce [11]. This contrasts with classic risk factor studies, where the outcome prevalence is routinely adjusted for covariates or confounders. In the case of infections with *Echinococcus*, covariates may include age or co-infection with other parasites such as *Taenia* spp.

Whereas the classical Hui-Walter model is based on the assumption of different populations which differ in their prevalences, in practice, it could be difficult to justify the splitting of one population into sub-populations. The separation of a population into different “prevalence populations” based on a factor which might interact with one of the tests (e.g., age or co-infection with another pathogen) is questionable [4], since sensitivities and specificities might not be constant in these different populations. Including a covariate instead offers in addition the assessment if this covariate is significantly associated with the prevalence and this association can be quantified in terms of an odds ratio.

The aims of this study were to obtain test accuracy and prevalence estimates for the diagnosis of *Echinococcus granulosus* and *Echinococcus multilocularis* in dogs in a highly endemic district of Sichuan province on the eastern Tibetan plateau. Three different tests were used for the diagnosis of *E. granulosus* and *E. multilocularis* infections in dogs, i.e. a genus-specific copro-antigen test, two species-specific copro-PCRs (one for each species of *Echinococcus*), and arecoline purgation. The results of these diagnostic tests on this population of dogs were used to estimate the diagnostic sensitivities and specificities of the tests using latent-class analysis. Age, sex and *Taenia* spp co-infection have been integrated as covariates in the latent-class models and their effects on the true prevalence have been assessed. The types of tests used and the nature of the data collected allowed for a full latent-class analysis. The use of covariates in the analysis and appropriate prior assumptions on the specificity of arecoline purgation enabled us to explore the dependence structure of these tests in this population of dogs. In addition, because we had parasite abundance data from the results of arecoline purgation, we were able to explore the hypothesis that the intensity of infection with *Echinococcus* spp affected the diagnostic sensitivity of copro-antigen ELISA and copro-PCR tests.

## Material and Methods

### Study area and animals

A total of 365 dogs from a highly *Echinococcus*-endemic region of the Eastern Tibetan Plateau in the People's Republic of China were sampled. Full details of the study animals and study area can be found in previous publications [12], [13]. Dog fecal samples were collected, and dogs subsequently received treatment, if their owners consented. Because the sampling was non-invasive, no prior ethical permission was sought. A table with data on test results classified according to *Taenia* co-infection is available in the supplementary online file (Table S1).

### Diagnostic tests

#### Copro-antigen ELISA

Faecal samples were collected during arecoline testing and stored in 5% formal saline with 0.3% tween 20. Faeces were tested in an *Echinococcus*-genus specific copro-antigen ELISA according to [14].

#### Species-specific copro-PCRs

Dogs were tested for *E. granulosus* using the method detailed by [15] and for *E. multilocularis* using a PCR according to [16]. Faecal samples were collected at the time of arecoline purgation and fixed in 95% ethanol.

#### Arecoline purgation

This procedure has been described in detail in previous reports [12], [13].

### Statistical analysis

A Bayesian approach was used to obtain estimates for the test accuracies of the three tests. Initial analyses with non-informative priors as beta distributions (1, 1) were used for all parameters, except for the faecal counts of adult parasites following purge, where the specificity was set at 1. This was justified as all purge positive samples had been confirmed morphologically through microscopic examination. Conditional dependencies between tests were assessed by separately examining the impact of each of the 4 covariance terms. In the case of three tests with unknown sensitivities and specificities, three pairs of covariance terms are possible (between tests 1 and 2, tests 1 and 3 and tests 2 and 3) for both sensitivity and specificity. Fixing one test specificity to 1 results in two covariance terms becoming obsolete, since if one test has a specificity = 1, then the test specificities of the two other tests must be conditionally independent from the first test. Models allowing for age, sex or *Taenia* spp co-infection to be a covariate for prevalence were tested. In addition it was possible to examine the performance of the tests by fixing the specificity of PCR to 1 instead of and/or in addition to fixing the specificity of purge to 1. Model selection was performed by using the deviance- information criterion (DIC) [17]. The DIC is used as a criterion for goodness of fit of the model. Smaller DIC, with a difference of at least 2 indicate a better fit of the model.

For each model, the first 20 000 iterations were discarded as burn-in and the next 50 000 iterations were used to parameterize the model. Multiple chains were run from different initial starting points and checked for convergence. Models were fitted with the software JAGS (http://mcmc-jags.sourceforge.net/) version 2.2.0, the software R (R, 2010) and the package coda. The model code is given in the supplementary online material (Text S1). To explore the possibility that the intensity of *Echinococcus* infection affected the diagnostic sensitivity of other tests we also undertook the analysis after reclassifying the results of the arecoline purgation. Therefore two further analyses were undertaken. When purge results indicated that the intensity of infection was less than 20 parasites, the purge results were classified as negative and the analysis repeated. For the second analysis reclassification was undertaken when purge results indicated a parasite intensity of between 1 and 99 parasites.

## Results

**Figure 1:**
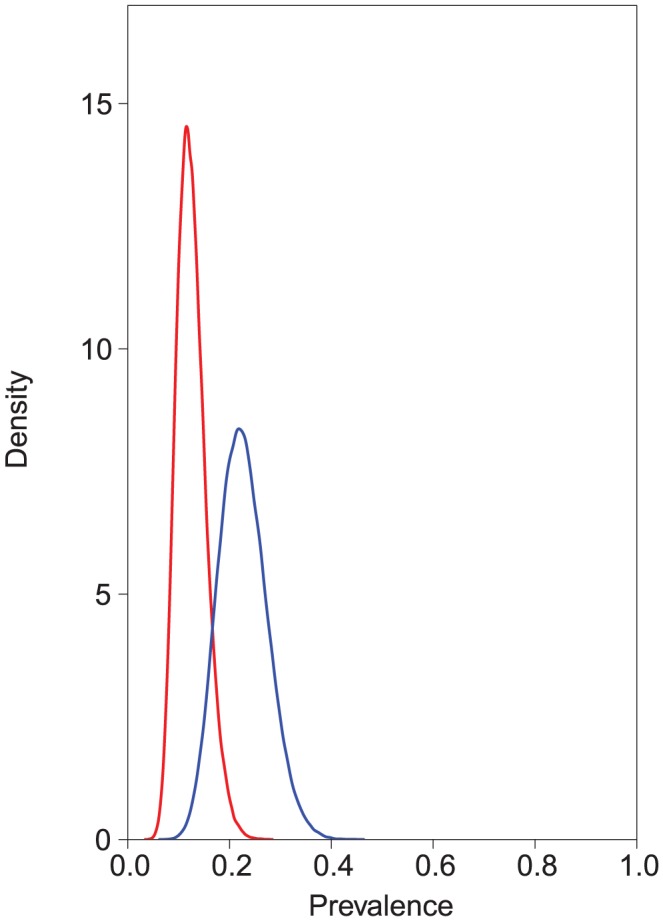
Effect of *Taenia* co-infection on prevalence of *E. multilocularis.* Using a Bayesian latent-class model, prevalence of *E. multilocularis* in *Taenia* test-negative dogs was estimated at 12.2% (95% credible intervals 7.6–18.9%), and in *Taenia* test-positive dogs was estimated at 22.3 (95% credible intervals 8.2–47.7%). *Taenia* co-infection was found to be a significant covariate with *E. multilocularis* infection (odds ratio 2.06, 95% credible intervals 1.07–3.9). In the figure the posterior distributions of the prevalence of *E. multilocularis* with (blue) and without (red) *Taenia* co-infection are shown.

**Figure 2:**
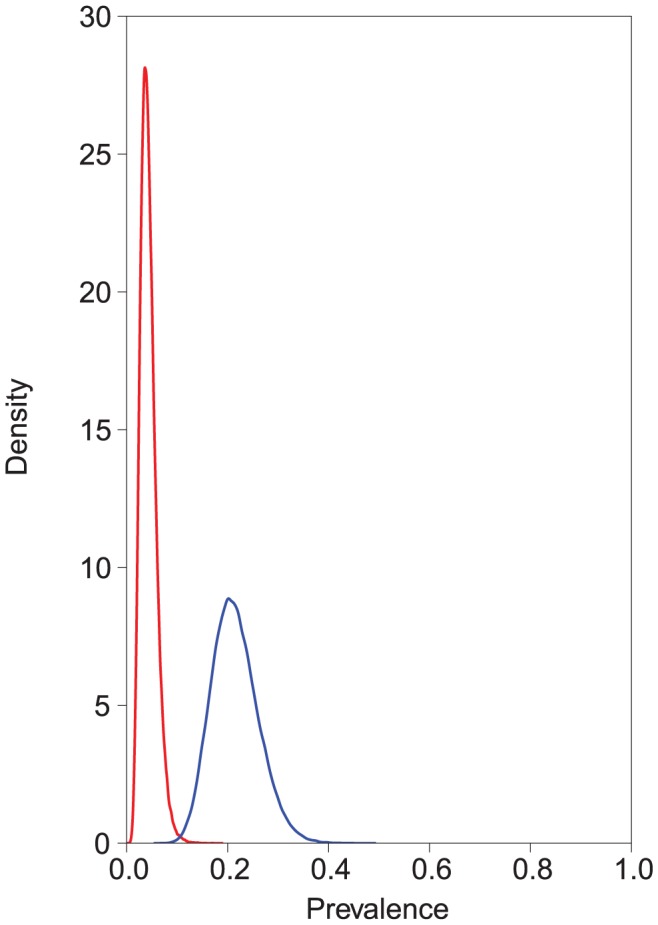
Effect of *Taenia* co-infection on prevalence of *E. granulosus.* Using a Bayesian latent-class model, prevalence of *E. granulosus* in *Taenia* test-negative dogs was estimated at 4.1% (95% credible intervals 1.9–8%), and in *Taenia* test-positive dogs was estimated at 21.1% (95% credible intervals 5.1–56.9%). *Taenia* co-infection was found to be a significant covariate with *E. granulosus* infection (odds ratio 6.32, 95% credible intervals 2.8–15.2). In the figure the posterior distributions of the prevalence of *E. granulosus* with (blue) and without (red) *Taenia* co-infection are shown.

The estimated test accuracies given as posterior means and their corresponding 95% credible intervals are presented in table 1 and the posterior density distributions in the figures S1, S2, S3, S4. In a Bayesian context, the results are given as posterior density or probability distributions which reflect, given the data and prior information, what would be the most probable parameter values. The reported results have the lowest DIC of a number of competing model estimates. A better model fit was obtained by including a covariance term for a conditional dependence between the sensitivities of the copro-antigen ELISA and the copro-PCR for *E. multilocularis*. The true prevalence of *E. multilocularis* infection in this population of dogs was estimated at 15.3% (95% credible intervals 10.3–21.8%) and the prevalence of *E. granulosus* was estimated at 11.1% (95% credible intervals 6.7–20.1%) without *Taenia* co-infection included as a covariate in the model. *Taenia* co-infection was a significant covariate with both *E. multilocularis* infection (odds ratio 2.06, 95% credible intervals 1.07–3.9) and *E. granulosus* infection (odds ratio 6.32; 95% credible intervals 2.8–15.2) (figures 1 and 2). The prevalence of *E. multilocularis* in *Taenia* test-negative dogs was estimated at 12.2% (95% credible intervals 7.6–18.9%), and in *Taenia* test-positive dogs was estimated at 22.3 (95% credible intervals 8.2–47.7%). The prevalence of *E. granulosus* in *Taenia* test-negative dogs was estimated at 4.1% (95% credible intervals 1.9–8%), and in *Taenia* test-positive dogs was estimated at 21.1% (95% credible intervals 5.1–56.9%).

**Table 1:** Estimated test accuracies for *E. multilocularis* and *E. granulosus*.

Test	Specific for	Sensitivity (95% CI)	Specificity (95% CI)
ELISA	Species E.m.	55 (40.8;68.9)	70.6 (65.3;76.7)
	Species E.g.	56.9 (40;73)	69.3 (64.1;74.3)
	Genus *Echinococcus*	55.7 (44.6;66.6)	73.7 (68;79)
PCR	Species E.m.	89.2 (78.9;96.3)	92.8 (88.2;97.9)
	Species E.g.	83.8 (69.1;94.3)	82.9 (78.3;87.2)
Arecoline purgation	Species E.m.	75.8 (54.9;94.2)	1
	Species E.g.	55 (40.8;68.9)	1

Estimated test accuracies (posterior means) for *E. multilocularis* and *E. granulosus* and their 95% credibility intervals in the final model with *Taenia* co-infection as a covariate on prevalence.

Changing the cut-off for being classified as positive in the purge from at least one parasite detected to at least 20 or 100 parasites did affect the estimates of the sensitivities of the three tests differentially. The sensitivity of the purge decreased by a maximum of 13.9% (*E. multilocularis*) and 33.6% (*E. granulosus*). The sensitivity of the PCR decreased less by maximally 6.9% for *E. multilocularis* and 4.5% for *E. granulosus*. In contrast to this, the sensitivity of the copro-antigen ELISA increased by approx. 10% for both *E. granulosus* and *E. multilocularis*. The specificities for both *Echinococcus* species for the ELISA remained virtually the same and for the PCR decreased marginally by 6.6% for *E. multilocularis* and 2.9% for *E. granulosus*. Figures S5 and S6 show the effect of increasing the cut-off for a faecal sample to be classified as positive from at least 1 to at least 20 parasites detected. Increasing the cut-off leads to an increase in the posterior distribution of the sensitivity by approx. 10%. Results including the corresponding intervals are presented in table 2.

**Table 2:** Estimated test accuracies with different cut-offs of the arecoline purgation.

Test	Cut-off	Specific for	Sensitivity (95% CI)	Specificity (95% CI)
ELISA	≥20	Species E.m.	65.1 (48;80.6)	70.5 (65.3;75.5)
	≥100	Species E.m.	64.2 (45.4;81.2	69.9 (64.7;75)
	≥20	Species E.g.	67.2 (46.3;84.8)	69.7 (64.5;74.9)
	≥100	Species E.g.	74.3 (48.3;93.5)	69.5 (64.2;74.9)
PCR	≥20	Species E.m.	86.3 (72.1;96)	87.6 (83.1;92.1)
	≥100	Species E.m.	82.3 (64.7;94.7)	86.2 (81.4;91.1)
	≥20	Species E.g.	83.3 (63.7;96.2)	81.5 (76.6;86.1)
	≥100	Species E.g.	79.3 (51.3;97)	80 (75;84.8)
Arecoline purgation	≥20	Species E.m.	70.5 (46.6;92.5)	1
	≥100	Species E.m.	61.2 (35.2;88.1)	1
	≥20	Species E.g.	61.9 (33.9;94.6)	1
	≥100	Species E.g.	42.2 (14.9;88.1)	1

Estimated test accuracies (posterior means) for *E. multilocularis* and *E. granulosus* and their 95% credibility intervals with different cut-offs of the arecoline purgation for being classified as positive (at least 20 or 100 parasites).

Fixing the specificity = 1 of the copro-PCR instead or in addition to the specificity of the purge did not affect the specificity of the copro-antigen ELISA. However, the sensitivities of the copro-antigen ELISA and the purge decreased (10 to 50%) (table 3). However, the deviance-information criteria indicated that this model was a poorer fit than allowing the specificity of the copro-PCR to vary. Sex was not a significant covariate in any analysis indicating the true prevalence of *Echinococcus* spp infection did not vary between male and female dogs (data not shown).

**Table 3:** Estimated test accuracies with specificities of copro-PCR alone or with purge fixed to 1.

Test	Specificity fixed to 1	Specific for	Sensitivity (95% CI)	Specificity (95% CI)
ELISA	PCR	Species E.m.	46.3 (35.5;57)	70.3 (64.9;75.5)
	PCR + purge	Species E.m.	46.1 (35.4;57.2)	70.4 (64.9;75.6)
	PCR	Species E.g.	39.4 (29.4;49.4)	68.9 (63.1;74.4)
	PCR + purge	Species E.g.	39.1 (29.3;49.3)	69 (63.1;74.6)
PCR	PCR	Species E.m.	91.4 (81.1;98.3)	1
	PCR + purge	Species E.m.	46.1 (35.4;57.2)	1
	PCR	Species E.g.	90 (74.4;99.5)	1
	PCR + purge	Species E.g.	84.2 869.9;94.6)	1
Arecoline purgation	PCR	Species E.m.	53.6 (42.6;64.6)	99.3 (97.9;1)
	PCR + purge	Species E.m.	53.8 (42.7;64.6)	1
	PCR	Species E.g.	29.9 (20.9;39.3)	98.9 (97;99.9)
	PCR + purge	Species E.g.	30.1 (21;40.2)	1

Estimated test accuracies (posterior means) for *E. multilocularis* and *E. granulosus* and their 95% credibility intervals if the specificity of the copro-PCR alone or in addition to the specificity of the purge is fixed to 1.

## Discussion

This study on the diagnosis of canine echinococcosis has used latent-class modeling to estimate the true prevalence and the diagnostic test performances of three tests for each *Echinococcus* spp. Arecoline purgation is a test that has been widely used in the past such as in the *Echinococcus*-elimination campaign in New Zealand [18] and for some transmission studies in central Asia [2], [19]. One previous study that used latent-class analysis suggested that the sensitivity of arecoline purgation was poor, perhaps as low as 38% and 21% for the diagnosis of *E. granulosus* and *E. multilocularis* infection respectively [2]. In the present study, the best fitting model (covariance with *Taenia* infection) suggested the sensitivity of arecoline purgation was much higher (table 1) with a sensitivity of over 75%. The specificity of the PCR test for the diagnosis of both parasitic infections converged on a lower value in the present study then in [2] where it was estimated as being 93% and 100% respectively. The two copro-PCR tests were not the same: the former study relied on egg isolation followed by PCR whereas the present studies omitted the egg isolation stage. However, it is important to reconcile these major differences. In the former study, there were only two tests used and the ability of the dog to roam as opposed to being tied all the time was a significant covariate. If the PCR test is fixed with a specificity of 100%, then the performance of the arecoline purgation drops markedly and is more similar to the values described in [2] (table 3). This indicates that the estimates of the performance of the arecoline seem to be highly dependent on the models' ability to classify PCR positives as true positives or allow for some false positives. The latter are important as there are a number of animals in both studies that are purge negative but copro-PCR positive. Indeed when the PCR tests were first developed by [15], the specificity was estimated at 100%. However this estimate was based on a sample of 10 dogs from non-endemic areas. In a naturally infected population false positive PCR results may occur due to coprophagia of faeces containing *Echinococcus* eggs. Thus eggs ingested in this manner might passage the intestine resulting in a positive PCR result indistinguishable from a result generated from parasite material coming from an established infection.

It should also be considered that the diagnostic performance may vary with the population of dogs. For example, the mean number of *E. multilocularis* parasites recovered from each dog in the present study is 131 (95% CI 62–375) [13], [14]. This is significantly higher than the mean number of 65 (95% CI 22–123) parasites recovered by purgation from the study by Ziadinov et al [2] that reports the much lower sensitivity of purgation. It is therefore possible that the dog populations from the two studies had substantially different parasite abundancies. In addition, when we reclassified the diagnostic test results as being only positive if there were at least 20 or 100 parasites, arecoline purgation was considerably less sensitive. Thus a higher mean abundance in the Tibetan population of dogs compared to the Kyrgyzstan population of dogs might also partly explain the considerable variation in the sensitivity of arecoline purgation between the two studies. When we reclassified the diagnostic test results as being only positive if there were at least 20 or 100 parasites, the sensitivity of the copro-antigen ELISA increased by approx.10% for both *Echinococcus* species. This might be explained by the ELISA performing better with higher parasite abundance in faecal samples. Previous studies have suggested that the sensitivity of copro-antigen increases as the intensity of infection increased [20]. There is little variation in the sensitivity of the PCR test regardless of which scenario is studied, indicating a sensitivity of approximately 89% for the diagnosis of *E. multilocularis* and 84% for the diagnosis of *E. granulosus*. This appears to be somewhat more sensitive than the test described in [2]. However, the previous test could only diagnose patent infections as it relied on prior egg isolation from the faeces. For patent infections the two tests are more comparable with the previous test able to detect an estimated over 87% and 72% for *E. granulosus* and *E. multilocularis* respectively. In another study with three tests based on antigen detection, DIC was also used as model selection criteria and indicated the same “best” models as likelihood ratio tests [21].

This analysis failed to find age of dog as a significant covariate and hence concluding that prevalence of *Echinococcus* infection does not vary significantly with age. This is consistent with the previous analysis [12], although age may affect abundance of *E. granulosus* in this group of dogs [13]. Relatively lower parasite abundances in older canids has been suggested to be a result of exposure to the parasite and immunity to reinfection or by variations in infection pressure with age [5] and changes in abundance may not accompanied by changes in prevalence.

The significance of *Taenia* as a covariate is to be expected as dogs are infected with both *Taenia* and *Echinococcus* spp through predation and local prey species are infected with metacestodes from both genera of parasites. In the previous analysis of this set of purge and faecal samples, significant correlations of abundance of *Taenia* and *Echinococcus* spp were found [12]. However, the present analysis failed to identify dog sex as a significant covariate. This is in contrast to a previous analysis of the same data using logistic regression and assuming that the results of arecoline purgation were definitive [12] which suggested that the prevalence in male dogs was higher than in females. The present study examined dog sex as a covariate in the latent-class analysis of diagnostic test performance and hence included the sensitivity of the arecoline purgation in the analysis. Thus a number of dogs in the previous study would have been misclassified and would have affected the results of the regression analysis. Techniques are now becoming available to incorporate the latent but unknown infection status in regression analysis [22] and these should be used where possible to avoid reaching inappropriate conclusions about the possible significance of covariates in epidemiological studies.

In conclusion the results of this study demonstrate how the unknown true prevalence of *Echinococcus* spp in dogs can be estimated if a number of diagnostic tests are used in parallel with a suitable covariate structure. It also demonstrates that an identical diagnostic test may have a considerable difference in performance between different study populations. Sensitivity and specificity are population-dependent [23] and the terminology of “intrinsic diagnostic test characteristics” implying that these are “constant and universally applicable” across populations should be discouraged [24]. Thus multiple tests should ideally be used routinely in the population of interest if no perfect gold standard is available. In contrast to (formerly) used approaches like Kappa tests to assess agreement of test results beyond chance, Bayesian latent-class approaches are more suitable to model the prevalence and associated influencing factors in a robust way. Finally using the true prevalence rather than the test prevalence may give different results with regard to the importance of determinants (such as *Taenia* in the case of this data set) which are associated with infection. This is due to misclassification errors following false positive or false negative test results when using test results in a deterministic manner.

## Supporting Information

Figure S1 **Density distributions for sensitivities for the three diagnostic tests for** ***E. multilocularis.*** Posterior distributions of the sensitivities of three diagnostic tests estimated using a Bayesian latent-class model and fixing the specificity of the purge equal to 1 (red = ELISA, blue = PCR, green = arecoline purgation).
(EPS)

Figure S2 **Density distributions for specificities for the three diagnostic tests for** ***E. multilocularis.*** Posterior distributions of the specificities of two diagnostic tests estimated using a Bayesian latent-class model and fixing the specificity of the purge equal to 1 (red = ELISA, blue = PCR).
(EPS)

Figure S3 **Density distributions for sensitivities for the three diagnostic tests for** ***E. granulosus*** **.** Posterior distributions of the sensitivities of three diagnostic tests estimated using a Bayesian latent-class model and fixing the specificity of the purge equal to 1 (red = ELISA, blue = PCR, green = arecoline purgation).
(EPS)

Figure S4 **Density distributions for specificities for the three diagnostic tests for** ***E. granulosus*** **.** Posterior distributions of the specificities of two diagnostic tests estimated using a Bayesian latent-class model and fixing the specificity of the purge equal to 1 (red = ELISA, blue = PCR).
(EPS)

Figure S5 **Effect of increasing the purge cut-off on the sensitivity of the ELISA for** ***E. multilocularis.*** Posterior distributions of the sensitivity of the ELISA for *E. multilocularis* with two different cut-offs: increasing the cut-off for being classified as positive from at least 1 (red) to 20 parasites (black) in arecoline purgation results in an increase in sensitivity of the ELISA of approx. 10%.
(EPS)

Figure S6 **Effect of increasing the purge cut-off on the sensitivity of the ELISA for** ***E. granulosus.*** Posterior distributions of the sensitivity of the ELISA for *E. granulosus* with two different cut-offs: increasing the cut-off for being classified as positive from at least 1 (red) to 20 parasites (black) in arecoline purgation results in an increase in sensitivity of the ELISA of approx. 10%.
(EPS)

Table S1 **Results** **obtained by three different diagnostic tests classified according to** ***Taenia*** **spp. infection status.**
(DOC)

Text S1 **Code for Bayesian latent-class model with** ***Taenia*** **co-infection as a covariate on prevalence.**
(DOC)
