# Supplementary material for: Latent-Class Methods to Evaluate Diagnostics Tests for Echinococcus Infections in Dogs
Source: PLoS Negl Trop Dis. 2013 Feb 14;7(2):e2068. doi: 10.1371/journal.pntd.0002068 (PMC3573084; doi:10.1371/journal.pntd.0002068)
Supplement: Table S1 — Results obtained by three different diagnostic tests classified according to Taenia spp. infection status. (DOC) [file pntd.0002068.s007.doc]

Table 1: Results obtained by three different tests for the detection of *E. granulosus* and *E. multilocularis* classified of samples classified according to *Taenia* spp. infection status

| *E. granulosus* | | *E. multilocularis* | | Tests | | |
| --- | --- | --- | --- | --- | --- | --- |
| *Taenia* + | *Taenia -* | *Taenia* + | *Taenia -* | ELISA | PCR | Purge |
| 52 | 135 | 54 | 151 | - | - | - |
| 2 | 2 | 1 | 15 | - | - | + |
| 7 | 36 | 6 | 0 | - | + | - |
| 6 | 4 | 6 | 11 | - | + | + |
| 30 | 61 | 25 | 55 | + | - | - |
| 0 | 0 | 1 | 1 | + | - | + |
| 4 | 11 | 7 | 7 | + | + | - |
| 12 | 3 | 13 | 12 | + | + | + |
| Total: 113 | Total: 252 | Total: 113 | Total: 252 |  |  |  |

(+) indicates a positive test result, (-) indicates a negative test result
