# Supplementary material for: Latent-Class Methods to Evaluate Diagnostics Tests for Echinococcus Infections in Dogs
Source: PLoS Negl Trop Dis. 2013 Feb 14;7(2):e2068. doi: 10.1371/journal.pntd.0002068 (PMC3573084; doi:10.1371/journal.pntd.0002068)
Supplement: Text S1 — Code for Bayesian latent-class model with Taenia co-infection as a covariate on prevalence. (DOC) [file pntd.0002068.s008.doc]

Model description

♯ Each row in the data is a tested sample and the columns are the different test results, age sex and Taenia co-infection

♯ p represents the individual data points, q the different test result combinations from q[i,1] as +++ to q[i,8] as ---, pr the prevalence, s_1-3_ the different test sensitivities and c_1-3_ the test specificities, covsxy and covcxy are the conditional dependencies between two test sensitivities or specificities, m.gregg represents the name of the data set

##############################################################################################

var p[N], q[N,8], pr[N], L[N],checks[N,16];

♯ To define the individual parameters within the likelihood function. To model the eight probabilities following a multinomial distribution with a loop for each observation.

model {

for(i in 1:N){

q[i,1]<-pr[i]*(s1*s2*s3+covs12+covs13+covs23)+(1-pr[i])*((1-c1)*(1-c2)*(1-c3)+covc12+covc13+covc23);

q[i,2]<-pr[i]*(s1*s2*(1-s3)+covs12-covs13-covs23)+(1-pr[i])*((1-c1)*(1-c2)*c3+covc12-covc13-covc23);

q[i,3]<-pr[i]*(s1*(1-s2)*s3-covs12+covs13-covs23)+(1-pr[i])*((1-c1)*c2*(1-c3)-covc12+covc13-covc23);

q[i,4]<-pr[i]*(s1*(1-s2)*(1-s3)-covs12-covs13+covs23)+(1-pr[i])*((1-c1)*c2*c3-covc12-covc13+covc23);

q[i,5]<-pr[i]*((1-s1)*s2*s3-covs12-covs13+covs23)+(1-pr[i])*(c1*(1-c2)*(1-c3)-covc12-covc13+covc23);

q[i,6]<-pr[i]*((1-s1)*s2*(1-s3)-covs12+covs13-covs23)+(1-pr[i])*(c1*(1-c2)*c3-covc12+covc13-covc23);

q[i,7]<-pr[i]*((1-s1)*(1-s2)*s3+covs12-covs13-covs23)+(1-pr[i])*(c1*c2*(1-c3)+covc12-covc13-covc23);

q[i,8]<-pr[i]*((1-s1)*(1-s2)*(1-s3)+covs12+covs13+covs23)+(1-pr[i])*(c1*c2*c3+covc12+covc13+covc23);

♯ Error checking since (0,1) bounds could be exceeded which is not allowed as dealing with probabilities (if so, the parameter combination is allocated a vanishingly small likelihood value)

checks[i,1]<- s1*s2*s3+covs12+covs13+covs23;

checks[i,2]<- (1-c1)*(1-c2)*(1-c3)+covc12+covc13+covc23;

checks[i,3]<- s1*s2*(1-s3)+covs12-covs13-covs23;

checks[i,4]<- (1-c1)*(1-c2)*c3+covc12-covc13-covc23;

checks[i,5]<- s1*(1-s2)*s3-covs12+covs13-covs23;

checks[i,6]<- (1-c1)*c2*(1-c3)-covc12+covc13-covc23;

checks[i,7]<- s1*(1-s2)*(1-s3)-covs12-covs13+covs23;

checks[i,8]<- (1-c1)*c2*c3-covc12-covc13+covc23;

checks[i,9]<- (1-s1)*s2*s3-covs12-covs13+covs23;

checks[i,10]<- c1*(1-c2)*(1-c3)-covc12-covc13+covc23;

checks[i,11]<- (1-s1)*s2*(1-s3)-covs12+covs13-covs23;

checks[i,12]<- c1*(1-c2)*c3-covc12+covc13-covc23;

checks[i,13]<- (1-s1)*(1-s2)*s3+covs12-covs13-covs23;

checks[i,14]<- c1*c2*(1-c3)+covc12-covc13-covc23;

checks[i,15]<- (1-s1)*(1-s2)*(1-s3)+covs12+covs13+covs23;

checks[i,16]<- c1*c2*c3+covc12+covc13+covc23;

valid[i]<- step(1-q[i,1])*step(q[i,1])*

step(1-q[i,2])*step(q[i,2])*

step(1-q[i,3])*step(q[i,3])*

step(1-q[i,4])*step(q[i,4])*

step(1-q[i,5])*step(q[i,5])*

step(1-q[i,6])*step(q[i,6])*

step(1-q[i,7])*step(q[i,7])*

step(1-q[i,8])*step(q[i,8])*

step(1-checks[i,1])*step(checks[i,1])*

step(1-checks[i,2])*step(checks[i,2])*

step(1-checks[i,3])*step(checks[i,3])*

step(1-checks[i,4])*step(checks[i,4])*

step(1-checks[i,5])*step(checks[i,5])*

step(1-checks[i,6])*step(checks[i,6])*

step(1-checks[i,7])*step(checks[i,7])*

step(1-checks[i,8])*step(checks[i,8])*

step(1-checks[i,9])*step(checks[i,9])*

step(1-checks[i,10])*step(checks[i,10])*

step(1-checks[i,11])*step(checks[i,11])*

step(1-checks[i,12])*step(checks[i,12])*

step(1-checks[i,13])*step(checks[i,13])*

step(1-checks[i,14])*step(checks[i,14])*

step(1-checks[i,15])*step(checks[i,15])*

step(1-checks[i,16])*step(checks[i,16]);

♯ Define/compute the contribution to the likelihood for the i^th^ observation

L[i]<- equals(valid[i],1)*(

equals(m.gregg [i,1],1)*equals(m.gregg[i,3],1)*equals(m.gregg [i,5],1)*q[i,1]

+ equals(m.gregg [i,1],1)*equals(m.gregg[i,3],1)*equals(m.gregg [i,5],0)*q[i,2]

+ equals(m.gregg [i,1],1)*equals(m.gregg[i,3],0)*equals(m.gregg [i,5],1)*q[i,3]

+ equals(m.gregg [i,1],1)*equals(m.gregg[i,3],0)*equals(m.gregg [i,5],0)*q[i,4]

+ equals(m.gregg [i,1],0)*equals(m.gregg[i,3],1)*equals(m.gregg [i,5],1)*q[i,5]

+ equals(m.gregg [i,1],0)*equals(m.gregg[i,3],1)*equals(m.gregg [i,5],0)*q[i,6]

+ equals(m.gregg [i,1],0)*equals(m.gregg[i,3],0)*equals(m.gregg [i,5],1)*q[i,7]

+ equals(m.gregg [i,1],0)*equals(m.gregg[i,3],0)*equals(m.gregg [i,5],0)*q[i,8]

) +(1-equals(valid[i],1)) *(1e-14);

♯ This is a trick to allow bespoke likelihood definition – as per WinBugs manual

logit(pr[i])<-intercept+slope*m.gregg[i,15];

p[i] <- L[i] / 1;

ones[i] ~ dbern(p[i]);

}

♯ Define priors (which may be posterior distributions or fixed values)

covs12~dbeta(1,1);

covs13<-0;

covs23<-0;

covc12<-0;

covc13<-0;

covc23<-0;

c1~dbeta(1,1);

c2~dbeta(1,1);

c3<-1

s1~dbeta(1,1);

s2~dbeta(1,1);

s3~dbeta(1,1);

intercept~dnorm(0,0.001);

slope~dnorm(0,0.001);

logL<-sum(log(p[1:N]));

}
